# Case Report: From unresectable to resectable: successful conversion therapy with TACE, HAIC, envafolimab, and olaparib in advanced intrahepatic cholangiocarcinoma

**DOI:** 10.3389/fonc.2026.1935499

**Published:** 2026-10-12

**Authors:** Chi Ma, Kun Feng, Jun Zhou

**Affiliations:** 1 Department of Radiology, Qilu Hospital (Qingdao), Cheeloo College of Medicine, Shandong University, Qingdao, China; 2 Department of Pathology, Qilu Hospital (Qingdao), Cheeloo College of Medicine, Shandong University, Qingdao, China; 3 Department of Interventional Radiology, Qilu Hospital (Qingdao), Cheeloo College of Medicine, Shandong University, Qingdao, China

**Keywords:** conversion therapy, immunotherapy, intrahepatic cholangiocarcinoma, locoregional therapy, PARP inhibitors, PD-L1 inhibitors

## Abstract

**Background:**

Intrahepatic cholangiocarcinoma (iCCA) is an aggressive malignancy with poor prognosis, and most patients present with unresectable or metastatic disease. In recent years, approaches such as locoregional therapy, immunotherapy and targeted therapy have achieved significant breakthroughs in the transformation treatment of advanced iCCA. We report a case of successful conversion therapy using envafolimab plus olaparib combined with locoregional therapies in advanced iCCA.

**Case presentation:**

A 65-year-old male with obstructive jaundice was diagnosed with metastatic iCCA (cT3N1M1, Stage IV) harboring a 117 mm left hepatic lobe lesion, with enlarged lymph nodes, and pulmonary nodules suspicious for metastasis. Next-generation sequencing revealed MSI-H and TMB-H status, along with DDR gene alterations (CDK12 and HDAC2). He received multimodal treatment comprising TACE, HAIC, PD-L1 blockade (envafolimab), and exploratory PARP inhibition (olaparib). Over 20 months, the primary lesion shrank from 117 mm to 42 mm, with regression of lymph nodules and pulmonary nodules and normalization of CA19-9. He underwent R0 resection and pathological examination confirmed a pathological complete response (pCR/ypT0N0) in the resected specimens. At the last follow-up (October 15, 2025), the patient had achieved a progression-free survival of 36 months and a postoperative recurrence-free survival of 16 months.

**Conclusion:**

This case demonstrates that the TACE plus HAIC combined with PD-L1 blockade and exploratory PARP inhibition is a promising conversion therapy strategy. It provides preliminary evidence for PARP inhibitor therapy in DDR-altered cholangiocarcinoma, supporting further investigation in prospective trials.

## 1 Introduction

Intrahepatic cholangiocarcinoma is among the malignancies with the poorest prognosis, with a five-year survival rate below 10% (1). Most patients are diagnosed at an advanced stage due to its insidious symptoms, losing the opportunity for curative resection (2). For unresectable or metastatic disease, the gemcitabine plus cisplatin (GC) regimen has been the mainstay systemic chemotherapy for advanced iCCA for many years; however, the median overall survival (mOS) is only approximately 11.7 months (3), highlighting the urgent need for novel therapeutic strategies to improve survival and prognosis.

For patients with specific molecular alterations, immunotherapy- or targeted therapy-based strategies may offer additional treatment options. In recent years, most international guidelines recommend immune checkpoint inhibitors targeting PD-1/PD-L1 (such as durvalumab or pembrolizumab) combined with the GC regimen as first-line treatment, improving mOS to approximately 12.9 months (4, 5). Patients with deficient mismatch repair (dMMR) or microsatellite instability-high (MSI-H) derive significant additional benefit from pembrolizumab, with an objective response rate (ORR) of up to 40-50% (6, 7). Additionally, approximately 30–40% of biliary tract tumors harbor targetable genetic alterations (5), and preliminary studies of immunotherapy combined with targeted therapy in iCCA have shown promising therapeutic prospects.

With advances in interventional radiology, hepatic arterial infusion chemotherapy (HAIC) has shown great potential in the conversion therapy of advanced iCCA. The latest National Comprehensive Cancer Network (NCCN) Biliary Tract Cancer Guidelines have recently incorporated HAIC as a recommended option for selected patients with unresectable iCCA (8), reflecting the accumulating evidence and increasing clinical acceptance of HAIC in appropriate patients. However, reports on the efficacy and safety of HAIC in combination therapy (including targeted agents and immunotherapy) remain scarce.

Therefore, we report a case of advanced iCCA harboring MSI-H/high tumor mutational burden (TMB-H) and DDR gene mutations (CDK12 and HDAC2). The patient received a multimodal regimen including transarterial chemoembolization (TACE), HAIC, PD-L1 blockade, and exploratory PARP inhibitor therapy, which successfully reduced the tumor and enabled R0 resection, with pathological examination of the resected specimen confirming a pathological complete response(pCR/ ypT0N0).

## 2 Case presentation

A 65-year-old male presented with a three-month history of progressive abdominal distension and dull pain, followed by the recent onset of jaundice. The patient was admitted on September 28, 2022. Initial contrast-enhanced abdominal computed tomography (CT) and magnetic resonance imaging (MRI) showed a large left hepatic lobe mass (approx. 117 mm) with intrahepatic biliary dilation and visceral peritoneal penetration. Baseline abdominal CT (October 6, 2022) revealed enlarged lymph nodes with rim enhancement. Regional nodes included the right cardia (station 1), lesser curvature of the stomach (station 3), and hepatoduodenal ligament (station 12). Non-regional nodes included the supradiaphragmatic (cardiophrenic angle) region, infradiaphragmatic region (station 19), and periesophageal region (station 20). The two largest nodes were located at the right cardia (station 1, short-axis diameter approximately 25 mm) and the lesser curvature of the stomach (station 3, short-axis diameter approximately 27 mm). Chest CT identified four pulmonary nodules suspicious for metastasis: in the left lower lobe (9 mm), the right middle lobe adjacent to the oblique fissure (8 mm), the right lower lobe (7 mm), and the right upper lobe (6 mm). Laboratory investigations confirmed obstructive jaundice with markedly elevated total bilirubin (154.3 μmol/L) and tumor marker CA19-9 (217 U/mL). On September 30, 2022, the patient underwent endoscopic retrograde cholangiopancreatography (ERCP) with endoscopic sphincterotomy (EST) and plastic biliary stent placement to relieve the biliary obstruction.

A CT-guided liver biopsy was performed on October 17, 2022. Histopathological examination confirmed a diagnosis of adenocarcinoma. Immunohistochemical staining showed CK7 (focal +), CD10 (occasional cells +), CK19 (+), CDX-2 (+), TTF-1 (−), Napsin A (−), and Ki-67 (+, 60%), suggestive of biliary origin (**Figures 1A, B**). Molecular analysis of the biopsy tissue was performed using next-generation sequencing (NGS). Clinical staging was assigned as cT3N1M1 (Stage IV), with the M1 designation based on imaging findings and not pathologically confirmed.

**Figure 1:**
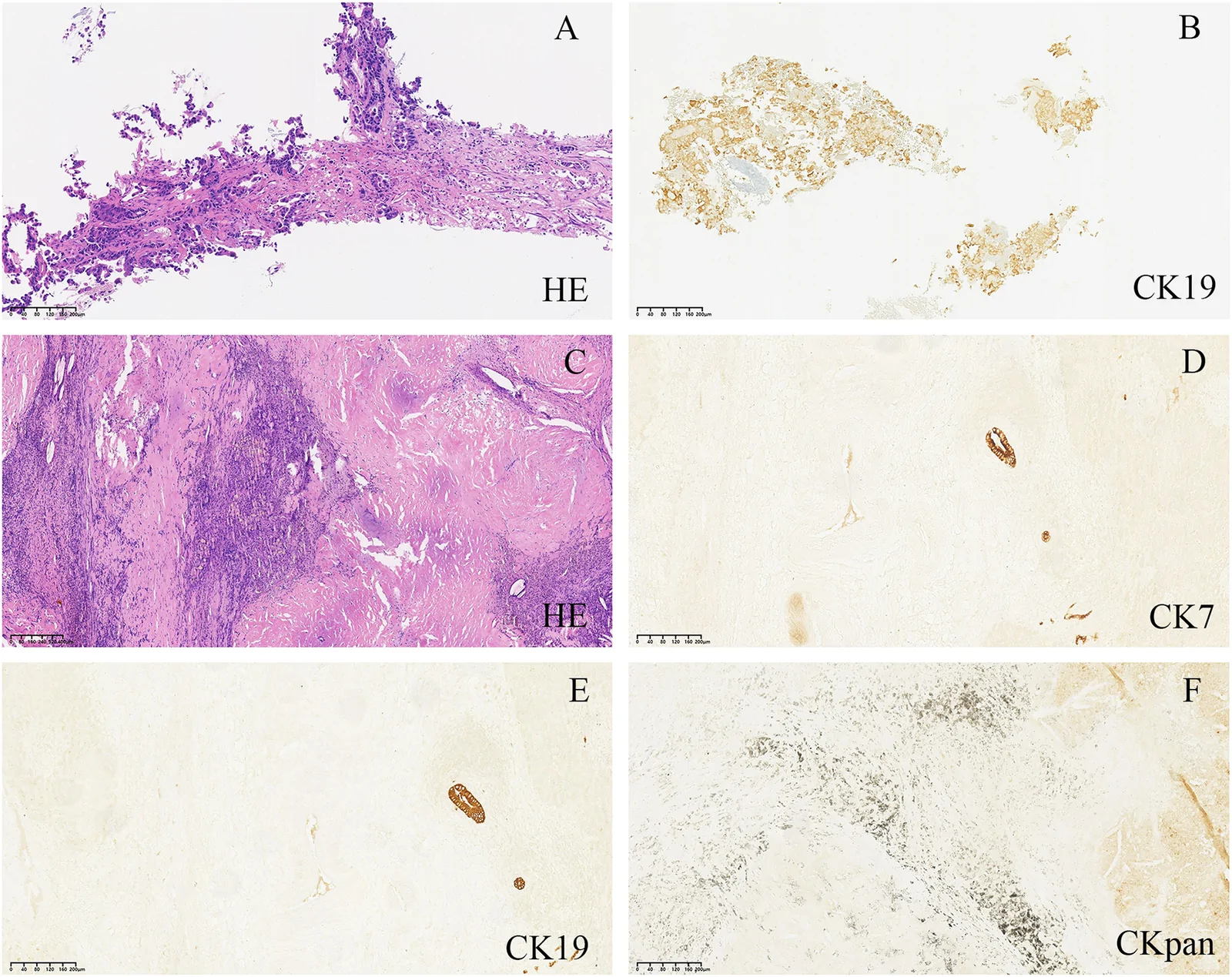
Pathological images of the liver tumor biopsy and postoperative specimens. **(A, B)** HE staining (10×) and CK19 staining (10×) of the liver tumor biopsy. **(C–E)** Representative HE (4×), CK7 (10×), and CK19 (10×) staining sections from tumor-bed areas. **(F)** CKpan staining (10×) of the sampled lymph node. (Scale bars are shown in the lower left corner of each image).

After MDT discussion, the patient was considered unsuitable for curative resection at presentation. Systemic chemotherapy or locoregional therapy (TACE plus HAIC) were both considered feasible options. Immunotherapy- and targeted therapy-based regimens were to be determined according to the NGS results. Due to the patient’s refusal of systemic chemotherapy, the MDT decided to proceed with TACE plus HAIC first for initial tumor control. The NGS report subsequently revealed an MSI-H/TMB-H phenotype (MSI score 0.69; TMB 76.6 Muts/Mb) with somatic class II alterations in MLH1, MSH2, MSH6, POLE, CDK12, HDAC2, and KRAS. MSI-H was defined as an MSI score ≥0.25 based on the assay’s validated threshold, and TMB-H was defined as ≥10 mutations per megabase (mut/Mb), consistent with the FDA-approved threshold. Germline testing revealed no pathogenic variants. MSI-PCR and MMR-IHC were not performed. Detailed assay parameters are provided in **Table 1**. After MDT reconvened, PD-L1 blockade was selected given the MSI-H/TMB-H phenotype. Olaparib was added on an exploratory, off-label basis given the DDR-related gene alterations (CDK12 and HDAC2) after informed consent.

**Table 1:** Summary of molecular profiling results.

Parameter	Result
MSI status	MSI-H (MSI score 0.69)
TMB	76.6 Muts/Mb (synonymous + non-synonymous); 60.99 Muts/Mb (non-synonymous only)
MSI-PCR or MMR-IHC	Not performed
Somatic variants (class II)	
KRAS	p.G12D, VAF 29.19%, missense, gain-of-function
MLH1	p.G606Vfs*7, VAF 41.37%, frameshift, predicted LOF
MSH2	p.A230Lfs*16, VAF 24.03%, frameshift, predicted LOF
MSH6	p.F1088Sfs*2, VAF 24.07%, frameshift, predicted LOF
CDK12	c.2610-1G>A, VAF 4.42%, splice-site, predicted LOF
HDAC2	p.V12Sfs*20, VAF 27.88%, frameshift, predicted LOF
POLE	p.K1550Nfs*12, VAF 6.6%, frameshift, predicted LOF
Germline variants	No pathogenic/likely pathogenic variants detected

Assay details: Targeted NGS panel (551 genes: entire exon regions of 530 genes and partial intron regions of 28 genes) on the Illumina NextSeq 550DX/NovaSeq 6000 platform; matched peripheral blood as normal control; tumor cell fraction 35%; mean sequencing depth 1750.4×; Q30 ratio 93.0%. MSI-H threshold: MSI score ≥0.25. TMB-H threshold: ≥10 Muts/Mb (FDA-approved). Variant calling included single nucleotide variants, small insertions/deletions, copy number variations, and rearrangements. LOF, loss-of-function; MSI, microsatellite instability; MSI-H, microsatellite instability-high; TMB, tumor mutation burden; VAF, variant allele frequency.

The treatment timeline was as follows:

October 19, 2022: First TACE was performed. A microcatheter was superselectively advanced into the tumor-feeding branches of the left hepatic artery and right inferior phrenic artery. The chemoembolic mixture consisted of lobaplatin 50 mg, idarubicin 10 mg, raltitrexed 4 mg, and 5 mL of poppyseed oil iodized emulsion, followed by supplemental embolization with gelatin sponge particles.

November 30, 2022: Second TACE with the same protocol was performed. Concurrently, HAIC was delivered as a single administration using the GC regimen: gemcitabine 1 g (50 mL/h for 1 hour) followed by cisplatin 40 mg (50 mL/h for 5 hours).

December 10, 2022: Systemic therapy with envafolimab (300 mg subcutaneously every 2 weeks) and olaparib (300 mg orally twice daily) was initiated and maintained without interruption until the perioperative period. Both were withheld before surgery (envafolimab 21 days; olaparib 7 days). No unplanned treatment interruptions occurred.

Serial radiographic follow-up demonstrated a remarkable and sustained treatment response over 20 months. On the restaging CT (April 26, 2024), the left lateral hepatic lobe was markedly atrophic, the left portal vein branch was poorly visualized, and the tumor involved the visceral peritoneum (liver capsule). The primary lesion had shrunk to 42 mm (**Figures 2A–D**), the two largest lymph nodes (stations 1 and 3) had shrunk to 9 mm and 5 mm in short-axis diameter (**Figures 2E–L**), respectively, and the two residual pulmonary nodules had shrunk to 2–3 mm (the other two nodules had resolved completely by November 28, 2022) (**Figure 3**). The remaining enlarged lymph nodes had shrunk or partially resolved. CA19–9 normalized, and the best response was partial response (RECIST v1.1). The preoperative staging was recorded as ycT3N1M1 (Stage IV), with the M1 designation remaining unconfirmed. After MDT consensus, the patient underwent 3D laparoscopic left hemihepatectomy with lymphadenectomy on June 1, 2024, based on the indeterminate, non-active pulmonary nodules and an adequate future liver remnant (>40% by CT volumetry).

**Figure 2:**
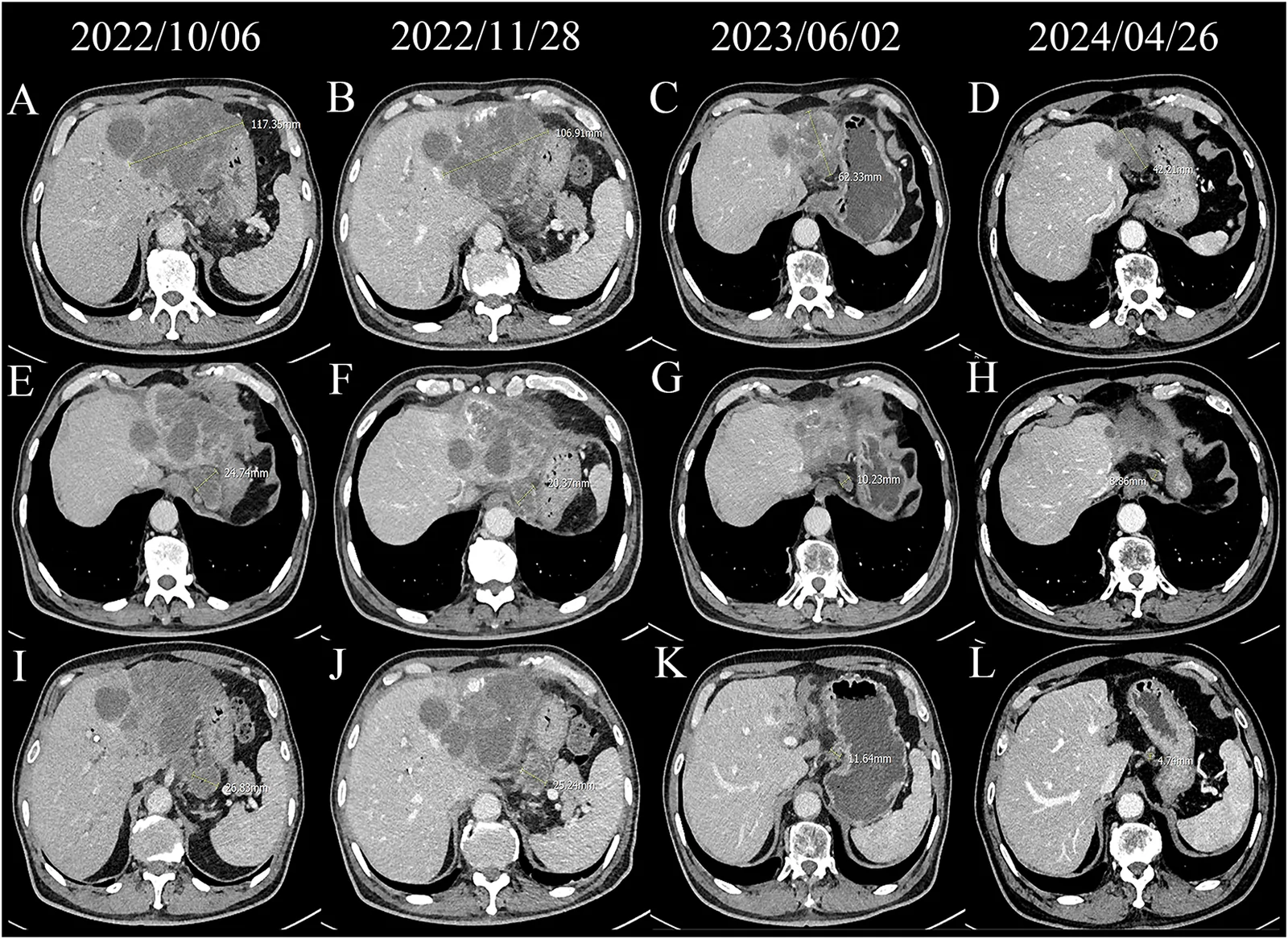
Radiological assessment of treatment response on CT imaging. **(A)** Initial contrast-enhanced CT scan (venous phase) reveals a large, hypodense mass lesion in the left hepatic lobe, measuring approximately 117 mm in maximum diameter. The mass had penetrated the visceral peritoneum, with obscuration of the left portal vein branch, suggestive of tumor thrombus. **(B–D)** Serial follow-up imaging illustrates progressive tumor regression. At 1 month after the first interventional procedure **(B)**, the lesion measured approximately 107 mm. Following two cycles of interventional therapy combined with immunotherapy and targeted therapy [7 months later, **(C)**], the tumor further decreased to 62 mm. Preoperative imaging **(D)** demonstrated continued shrinkage, with the lesion measuring approximately 42 mm. **(E-L)** Corresponding serial images of enlarged lymph nodes. The two largest target lymph nodes—at the right cardia (station 1) and lesser curvature of the stomach (station 3)—show a reduction in short-axis diameter from 25 mm and 27 mm at baseline to 9 mm and 5 mm, respectively, on the pre-surgical CT.

**Figure 3:**
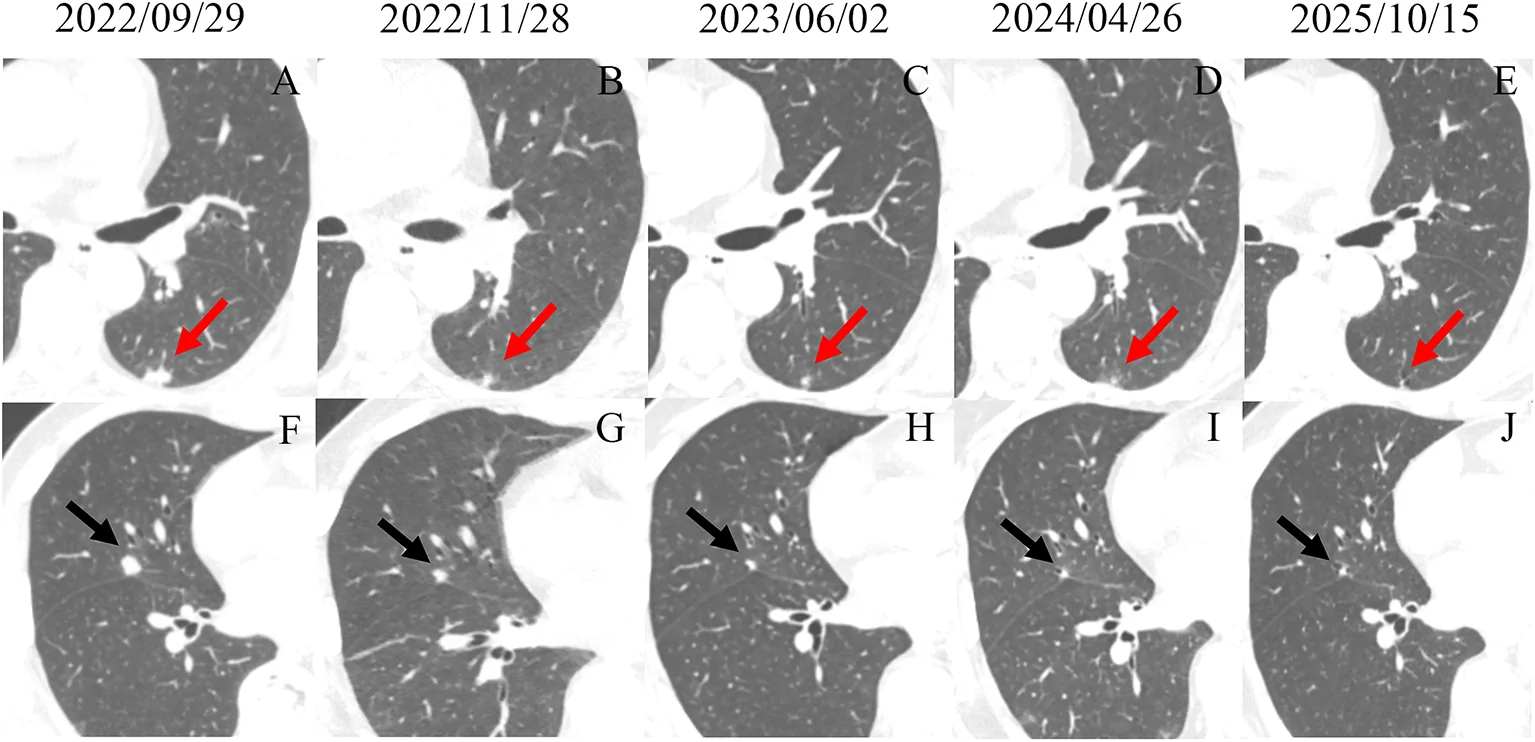
Computed tomography (CT) images demonstrate temporal regression of the two largest suspicious metastatic nodules during treatment. **(A–E)** Axial CT sections show a suspicious metastatic nodule in the left lower lobe (red arrow). **(F–J)** Corresponding images show a suspicious metastatic nodule in the right middle lobe (black arrow).

Intraoperatively, a left hepatic lobe tumor adherent to the diaphragm, abdominal wall, and stomach was identified, with enlarged stations 1 and 3 lymph nodes. No metastatic nodules were observed on the abdominal wall, pelvic cavity, greater omentum, or mesentery. Intraoperative lymphadenectomy included regional stations (1, 3, 8, 12) and non-regional stations (7, 9, 13, 16). Pathological examination, with extensive sampling of the tumor bed and surrounding liver parenchyma, revealed extensive fibrous proliferation with hyalinization, chronic inflammatory cell infiltration with multinucleated giant cell reaction, and focal necrosis, with no viable tumor cells. The liver margin was negative. Stations 1 and 3 lymph nodes showed treatment response (necrosis and fibrosis) with no residual tumor; no lymph nodes were identified in station 12. These findings are consistent with pCR/ypT0N0 (**Figures 1C–F**) in the resected specimens. As the pulmonary nodules were not excised or biopsied, systemic pCR could not be confirmed.

Safety assessment (CTCAE v5.0) showed no grade ≥3 adverse events. Grade 2 anemia and grade 1 leukopenia occurred on March 6, 2023, and resolved by April 28, 2023, with close observation, dietary modification, and oral iron supplementation; no dose adjustment was required. Grade 1 transient aminotransferase elevation, nausea, and fatigue also occurred without dose adjustment. No renal, immune-related, or injection-site adverse events occurred. The patient recovered well after surgery, with no postoperative complications. Envafolimab was discontinued postoperatively; olaparib was resumed 4 weeks after surgery as maintenance therapy for 3 months. Postoperatively, liver function and tumor markers were monitored every 1–2 months, with imaging as clinically indicated.

The patient was last formally followed up on October 15, 2025, at which time clinical examination and imaging confirmed no evidence of recurrence (**Figure 4**). The progression-free survival (PFS) from treatment initiation was approximately 36 months, the postoperative recurrence-free survival (RFS) was approximately 16 months. Subsequent telephone follow-ups have revealed no evidence of recurrence to date.

**Figure 4:**
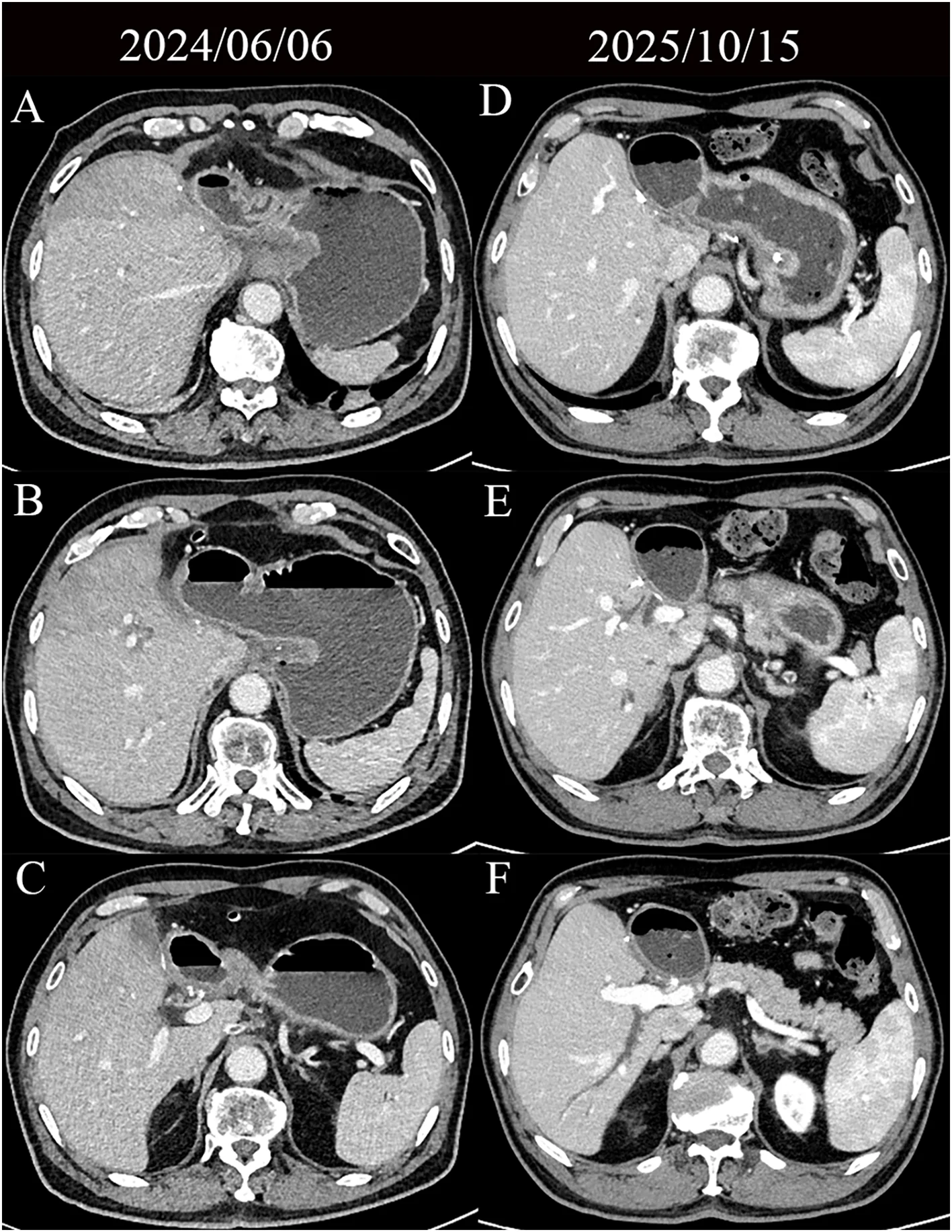
Postoperative CT imaging follow-up. **(A–C)** Initial postoperative contrast-enhanced CT scans (venous phase) demonstrate heterogeneous hepatic parenchymal enhancement. A hyperdense suture line (likely surgical material) and an adjacent linear, non-enhancing hypodense area (arrow) are seen along the resection margin, consistent with expected postoperative changes. **(D–F)** Recent follow-up CT scans (venous phase) show homogeneous hepatic parenchymal enhancement, with mild intrahepatic biliary dilation (interpreted as a benign postoperative compensatory change, given the absence of progression and negative tumor markers). There is no imaging evidence of tumor recurrence, metastasis, or lymphadenopathy.

## 3 Discussion

For unresectable advanced intrahepatic cholangiocarcinoma, the current therapeutic goal is to achieve tumor downstaging through conversion therapy—including locoregional approaches (e.g., TACE, HAIC, radiotherapy, and selective internal radiation therapy) and systemic approaches (e.g., chemotherapy, immunotherapy, and targeted therapy)—to convert unresectable disease into resectable disease (5).

HAIC involves inserting a catheter via minimally invasive methods into the arterial blood supply of liver tumors and continuously infusing high-concentration chemotherapy drugs. Due to first-pass metabolism, HAIC can achieve up to 400-fold higher intrahepatic drug concentrations than systemic chemotherapy, while reducing systemic exposure and adverse effects (9). For patients who cannot tolerate or decline systemic chemotherapy, HAIC is an alternative conversion therapy option. Existing evidence suggests that HAIC combined with immunotherapy and targeted therapy can achieve superior efficacy compared with systemic chemotherapy alone (10, 11), supporting the use of the TACE plus HAIC locoregional strategy in this case.

In this case, the MSI-H/TMB-H phenotype provided a strong rationale for PD-L1 blockade. The MSI-H state resulting from dMMR has been validated as an effective predictive biomarker for immune checkpoint inhibitor therapy across cancer types (6, 12). Envafolimab used in this case features a unique single-domain antibody structure. As the world’s first subcutaneously administered PD-L1 inhibitor, it demonstrates significant advantages in administration convenience and patient compliance. Completed phase II trial data (NCT03667170) show that envafolimab exhibits favorable safety and confirmed efficacy in patients with MSI-H/dMMR advanced solid tumors, with an objective response rate of 42.7% and a 12-month overall survival rate of 74.6% (13).

DNA damage repair (DDR) is a broad network comprising multiple repair pathways, of which HRR is a key pathway for repairing DNA double-strand breaks. PARP inhibitors exploit synthetic lethality to selectively kill tumor cells with HRR deficiency (14). Clinical evidence has demonstrated that patients with biliary tract cancers may benefit from PARP inhibitors, including a phase II(NCT04306367) trial of pembrolizumab plus olaparib in advanced BTC (15) and several case reports in BRCA-mutated iCCA (16, 17). However, PARP inhibitors have not yet been approved for cholangiocarcinoma.

In this case, CDK12 and HDAC2 are DDR-related genes but are not conventional core HRR genes (such as BRCA1/2). Preclinical studies have shown that CDK12 deficiency can downregulate BRCA1 expression and impair HR repair, thereby conferring sensitivity to PARP inhibitors (18). HDAC2 regulates RAD51 expression and affects HR repair, and its inhibition can enhance PARP inhibitor sensitivity (19). However, clinical evidence specific to CDK12 and HDAC2 in cholangiocarcinoma remains extremely limited. Therefore, the use of olaparib in this case was exploratory and off-label, administered after informed consent.

The response observed in this case may hypothetically reflect multi-layered effects: TACE and HAIC may release tumor antigens and trigger an *in situ* vaccine effect, creating favorable conditions for immunotherapy (20, 21); Envafolimab blocks the PD-1/PD-L1 pathway to relieve immunosuppression, transforming local inflammatory responses into systemic anti-tumor immunity (22); and Olaparib may activate cGAS-STING and enhance CD8+ T-cell infiltration, thereby converting an immunologically “cold” tumor into a “hot” one and potentiating the efficacy of PD-L1 blockade (23–25). However, these effects cannot be demonstrated from a single case and require validation in large-scale mechanistic studies.

In summary, this case suggests that TACE plus HAIC combined with PD-L1 blockade and exploratory PARP inhibition is a promising conversion therapy strategy, providing preliminary evidence for the use of PARP inhibitors in advanced iCCA patients with DDR gene mutations and warranting further investigation.

## 4 Limitations

Several limitations should be acknowledged. First, as a single case report, the findings are not generalizable, and the observed response cannot be attributed to any specific treatment component. Second, the MSI-H status was determined solely by NGS without confirmatory MSI-PCR (the current gold standard) or MMR-IHC, representing a methodological limitation. Third, the DDR pathway alterations identified (CDK12 and HDAC2) were not confirmed to be functionally deleterious, and their association with olaparib sensitivity has not been validated in cholangiocarcinoma; therefore, the rationale for PARP inhibition is exploratory rather than definitive. Fourth, the pulmonary lesions were not pathologically confirmed, and systemic pCR could not be verified. Fifth, no mechanistic studies (cGAS-STING pathway, CD8+ T-cell infiltration, or tumor microenvironment analyses) were performed, and the proposed contribution between treatment components remains speculative. Sixth, the HAIC GC regimen (gemcitabine 1 g and cisplatin 40 mg) was administered at half of the body-surface-area-calculated dose, based on the higher intrahepatic drug concentrations achieved with HAIC; however, HAIC regimens for cholangiocarcinoma remain non-standardized, and no unified international guideline for HAIC dosing currently exists. Seventh, the long-term survival benefit of this approach remains unknown, and the promising results observed require validation in larger prospective studies.

## Data Availability

The original contributions presented in the study are included in the article/supplementary material. Further inquiries can be directed to the corresponding author.
